# Training the trainers: an interview with Carol Ibe on the importance of building networks for agricultural research in African countries

**DOI:** 10.1038/s42003-021-02568-2

**Published:** 2021-09-09

## Abstract

Carol Ibe is a Postdoctoral Scientist in the Saunders Lab at the John Innes Centre, Norwich. She received her PhD in Plant Sciences (as a Gates Scholar) from the University of Cambridge in 2020. From Nigeria, Carol understands the urgent need to develop the right capacity to advance bioscience education, research, and innovation to eradicate hunger, malnutrition, and extreme poverty in Africa. This led her to set up the JR Biotek Foundation, a non-profit organisation that is providing Africa-based early-career agricultural researchers with the right skillset and opportunities to improve widely consumed crop varieties in African countries, and to foster links between the lab and market to support smallholder farmers in the region. Her unwavering dedication and passion to improve the lives of others through her research and Foundation’s work has led to numerous awards including the University of Cambridge Society for the Application of Research Awards (CSAR) and the 2019 Bill Gates Sr. Prize.

**Figure:**
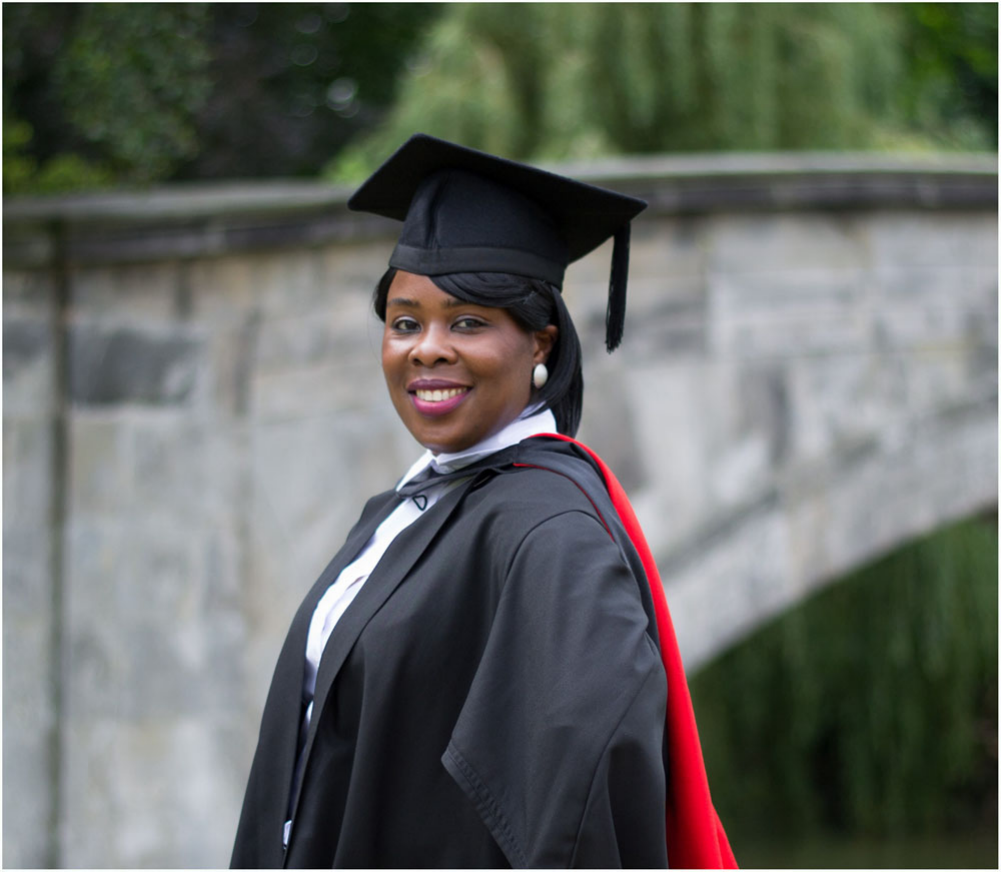
Jerelle A. Joseph

(1) Please tell us about your background and how it has motivated your research interests.

I grew up in Nigeria and did my undergraduate studies (in microbiology) there. I saw how food was often rationed in many households because it was simply not enough. People struggled (and still struggle) to make ends meet, and for the majority of the population, adequate nutritious food was a luxury and not a basic need. Sadly, this situation is mirrored across many parts of Africa, both in the urban and rural communities, and in as much as these problems plagued us, studying for a degree in agricultural science was unpopular because it was seen as an unattractive and non-lucrative career. Seeing my grandparents and some of our relatives struggle to earn a decent living on subsistence farming, which weighed heavily on their physical health, also put me off. I moved to the United States right after my undergraduate studies in Nigeria and studied for a master’s in molecular biology and biochemistry at Georgetown University. At Georgetown, I realised that my undergraduate degree did not adequately prepare me for the next steps in my science career. Whilst my peers were a hundred steps ahead, I was struggling to catch up. I was very disappointed by this setback and out of frustration, I thought of setting up an organisation that will equip science students in African countries with modern scientific skills that are practical and relevant to solving problems faced on the continent. After working in research for a few years and also obtaining a second master’s degree from the University of Oxford (UK), I started the JR Biotek Foundation, a non-profit organisation that is advancing bioscience education, research and innovation for Africa’s sustainable development.

My vision for the Foundation at the time was to help and build biomedical and agricultural research capacity in Africa; however, after organising and teaching our very first hands-on molecular biology and biotechnology workshop in Nigeria in September 2014, I noticed that over 80% of the workshop applicants and participants from different African countries were involved in agricultural research. I wondered why. After discussing with the workshop participants and doing some more research, I understood the urgent need for the application of modern molecular biology and biotechnology principles, laboratory techniques and research methodologies to improve agricultural productivity and sustainable food production in Africa. To be a part of the solution, I embarked on a journey as a PhD student in Plant Sciences at the University of Cambridge where I was a Gates scholar. My PhD focused on rice, a staple cereal and food security crop in Africa and Asia, and how its roots interact with a mutualistic fungus (Rhizophagus irregularis) and a deleterious pathogen (Magnaporthe oryzae). I uncovered a novel role of rice plasma membrane receptors in enabling M. oryzae invasion of rice roots, an extremely important finding that may lead to new thinking about how plant pathogens exploit the competitive and promiscuous nature of host receptors to invade their hosts. A deeper understanding of such plant-fungal communication processes will reveal new targets for crop improvement and strengthen global food security, especially in sub-Saharan Africa.

Presently, I am a postdoctoral research scientist in Diane Saunders Lab at the John Innes Centre in Norwich, United Kingdom. My overarching research aim is to build a better understanding of how the yellow rust pathogen, *Puccinia striiformis* f. sp. *tritici* (*Pst*) manipulates its wheat host, and to use this new knowledge to inform future disease management strategies. Pst causes yellow rust, a deleterious disease that leads to huge wheat yield losses and a major threat to wheat production worldwide. Like rice, wheat is also a major staple cereal for 40 percent of the world’s population, very high in demand, particularly in developing countries where 50 percent of annual global wheat production are harvested. In Africa, the demand for wheat is growing considerably due to the rapid population growth amongst other factors, therefore, developing new forms of disease resistance in wheat crops is urgently needed to sustainably increase wheat production in sub-Saharan Africa and globally. I do science for a reason and that reason is to make important findings that will make a real difference in the lives of others, and not just to climb the academic ladder.

(2) Could you tell us about your work and motivations in launching the non-profit JR Biotek Foundation?

My experience as a student at Georgetown motivated me to start the JR Biotek Foundation. The first day I stepped my foot into the laboratory, all I could think of was how to provide similar opportunities to students and early-career researchers (ECRs) in African universities, especially those that do not have access to well-equipped labs, good-quality training and opportunities to advance their research. These thoughts became a burden until I summoned the courage to start the organisation. Our Foundation’s work focuses on helping African nations to advance research and innovation to accelerate solutions for achieving food and nutrition security, human health and environmental sustainability. We do this by providing fully funded hands-on molecular bioscience laboratory training programs aimed at equipping agricultural ECRs across Africa with knowledge, skills and abilities to improve their research and teaching. Our courses cover a broad range of topics on plant molecular biology, genetics and genomics, molecular plant breeding, bioinformatics and research data analysis using various statistical methods. As you may know, African researchers, especially plant breeders, collect a lot of data from the field but find it very challenging to analyse the data because of lack of expertise and lack of access to modern data analysis tools. We are bridging this gap by mobilising a team of researchers and breeding experts (PhD students, postdoctoral researchers and Principal Investigators) from top world universities to teach the researchers how to use modern tools and methods to analyse scientific data.

During my PhD at Cambridge, I initiated the Reach & Teach Science in Africa program out of the urgent need to reach and provide more researchers and students in Africa with our world-class scientific laboratory training programs. Each time we put out a call for a workshop, we received close to 1000 applications for 20–100 places from very qualified candidates all across Africa. It was painful not to offer all of them a place to gain the hands-on learning experience and networking opportunities that they needed, but we did not have the capacity to meet these needs. After two successful workshops held at the Department of Plant Sciences, University of Cambridge (2017–2018), which involved a total of 33 outstanding researchers, mostly PhD students (fully funded) from Africa who were selected from over 1000 applicants, we decided it was time to run the workshops in African research institutions. This would enable us to meet them where they are. So, in 2019, we partnered with the University of Abomey-Calavi in Benin Republic (Wes Africa) to host our first Reach & Teach Science in Africa plant molecular bio-laboratory training workshop for 100 outstanding agricultural ECRs from 19 African countries. The workshop was taught by fantastic ECRs (PhD students and postdoctoral researchers) mainly from Cambridge University and funded by several initiatives including our scholarship Trusts, the Department of Plant Sciences, Cambridge-Africa Alborada, OpenPlant, Trinity College and the Global Challenges Research Fund (GCRF).

Although we could not travel to Africa to run more workshops in 2020 due to the COVID-19 pandemic, which caused school closures globally, but worst hit across Africa where online learning was not in existence for the most part, we started the virtual Reach & Teach Science in Africa program to provide an avenue for active learning for bioscience students and researchers in the region. This involved mobilising lecturers from top world research institutions to teach a range of topics focusing on molecular biosciences, crop pathology and breeding and bioinformatics. Recently, we commissioned some of our exceptionally gifted alumni and researchers in African research institutions to co-organise and facilitate the 2021 Reach & Teach Science in Africa plant molecular bio-laboratory training workshop organised in partnership with the Centre for Dryland Agriculture at Bayero University Kano, (Nigeria), Masinde Muliro University of Science and Technology (Kenya) and the CSIR-Crops Research Institute in Kumasi (Ghana). The workshops involved 80+ participants, facilitators and research support staff all from Africa. It was exhilarating to see African researchers taking the lead to train others, and this is exactly why I started the JR Biotek Foundation—to train the trainers, and as we do so, we are building powerful scientific networks and capacity for agricultural research in African countries. To date, we have reached about 250 agricultural ECRs through our face-to-face workshops, and thousands of others through our virtual Reach & Teach Science in Africa program. It is like a ripple effect. The more we reach (directly), the more they (our alumni) reach because over 60% of them are involved in teaching and/or supervising undergraduate and master’s students, with average class sizes of 250-450 per year. This is what I consider a major impact and potentially transformative initiative that can significantly improve Africa’s bioscience education at the tertiary education level.

(3) Based on your personal experience or through your work with the Foundation, what unique challenges do African scientists face and what steps can be taken to overcome these challenges?

African scientists working in African nations face many challenges, and whilst some are easily solvable, many are complex and will require concerted efforts from African governments, development partners and other key stakeholders. Based on our Foundation’s research (yet to be published), we have identified the lack of funding for scientific research and related activities as a number one constraint hampering high-quality research and innovation across Africa. Other pressing challenges include the lack of access to good quality scientific training and capacity building, poor infrastructure development, inadequate modern laboratory equipment and consumables needed to carry out the level of research needed to solve the plethora of problems faced on the continent.

With Africa’s population expected to double to 2.4 billion people by 2050, I am very concerned that many more people including children may find it difficult to cope without decent daily meals. Agriculture is a major driver of Africa’s economic development because it employs over 50% of the population, especially in rural areas where farming is a primary source of food, income and employment. Agricultural research and innovation can help tackle food and nutrition insecurity in Africa, but there is currently a severe lack of capacity on the continent. Compared to over 4000 researchers per one million people in the United States and the United Kingdom, Africa is estimated to have an average of 198 researchers per one million people (https://www.weforum.org/agenda/2017/05/scientists-are-the-key-to-africas-future/). We also know that many African universities currently do not have the basic resources and facilities needed to support the next generation of African scientists, and this chronic impoverishment has created a huge gap in scientific knowledge between Africa-based scientists and their counterparts in the global north. This sad situation limits many intelligent students and skilful researchers on the continent to compete globally and to contribute to the continent’s sustainable development.

To overcome some of these challenges, African governments and all key stakeholders must work together to increase investments in Research and Development across all sectors, particularly in agriculture and health care. From my experience and from the work of our Foundation, there is an inevitable need to transform the tertiary education system in many African nations to make it globally competitive, productive, and efficient in providing modern innovative teaching, creativity, and problem-solving skills to a workforce that can solve the most pressing problems facing the African people and continent. Without this, I fear that our countries in Africa will continue to rely heavily on the global north for aid and resources that they themselves can produce, limiting their own capabilities and those of future generations. My own contribution and that of our organisation (the JR Biotek Foundation) is to build a powerful network of skilled researchers and professionals in Africa and the diaspora who can work together to develop solutions aimed at solving specific problems, especially food and nutrition insecurity in African countries.

(4) What are some exciting new research findings or avenues that you find the most promising toward achieving food security?

There are so many exciting research avenues that can contribute to a sustainable increase in food production, access to food and poverty eradication on a global scale, and it is a bit tricky to choose one over another. I, however, find the genome editing and ‘omics’ technologies as well as speed breeding approaches very promising for crop improvement and achieving food security. Whilst the application of these modern technologies and research approaches have advanced in more developed nations, they are still largely inaccessible across Africa due to insufficient or lack of efficient funding mechanisms needed to support the level of infrastructure development and research capacity (among other factors) these technological approaches require. The question then remains, how can African nations achieve food and nutrition security if they do not put in place the right structures and systems to enable their researchers to develop and lead their own research agenda without heavily relying on solutions created for them by their counterparts in the global north? I know for a fact that there are exceptionally talented researchers in Africa who are doing great research to tackle some of these problems, but the majority of them are in international research centres because many national universities are grossly underfunded. In my opinion, strategic and adequate investments in national research institutions is the way forward for African nations to produce a skilled scientific workforce that can advance research and innovation for the continent’s sustainable development.

(5) Who inspires you and your work?

I have been very fortunate to work with excellent people and scientists from all walks of life. I have also faced challenges in my science career, and sometimes, it is difficult to know whether you are treated in certain ways because of where you come from. Nevertheless, I have chosen to focus on the positives and not the negatives, learning as much as I can to give back to society wherever I can. For me, science is more than just a career and a race to fame. It is personal on different fronts because it allows me to use my education, research and opportunities presented to me to improve the lives of others. Being able to give back to African scientists and nations through my research and Foundation’s work inspires me to work even harder to develop new solutions and avenues to end hunger, malnutrition and extreme poverty in Africa and globally. This is my vision and those who share a similar passion and interest, inspire me.

*This interview was conducted by Associate Editor Caitlin Karniski*.

